# Structural basis of omalizumab therapy and omalizumab-mediated IgE exchange

**DOI:** 10.1038/ncomms11610

**Published:** 2016-05-19

**Authors:** Luke F. Pennington, Svetlana Tarchevskaya, Daniel Brigger, Karthik Sathiyamoorthy, Michelle T. Graham, Kari Christine Nadeau, Alexander Eggel, Theodore S. Jardetzky

**Affiliations:** 1 Department of Structural Biology, Stanford University School of Medicine, Stanford, California 94305, USA; 2 Progam in Immunology, Stanford University School of Medicine, Stanford, California 94305, USA; 3 Sean N. Parker Center for Allergy Research at Stanford University, Stanford University School of Medicine, Stanford, California 94305, USA; 4 Department of Rheumatology, Immunology and Allergology, University Hospital Bern, Bern 3012, Switzerland; 5 Department of Clinical Research, University of Bern, Bern 3012, Switzerland; 6 Department of Medicine, Stanford University School of Medicine, Stanford, California 94305, USA

## Abstract

Omalizumab is a widely used therapeutic anti-IgE antibody. Here we report the crystal structure of the omalizumab–Fab in complex with an IgE-Fc fragment. This structure reveals the mechanism of omalizumab-mediated inhibition of IgE interactions with both high- and low-affinity IgE receptors, and explains why omalizumab selectively binds free IgE. The structure of the complex also provides mechanistic insight into a class of disruptive IgE inhibitors that accelerate the dissociation of the high-affinity IgE receptor from IgE. We use this structural data to generate a mutant IgE-Fc fragment that is resistant to omalizumab binding. Treatment with this omalizumab-resistant IgE-Fc fragment, in combination with omalizumab, promotes the exchange of cell-bound full-length IgE with omalizumab-resistant IgE-Fc fragments on human basophils. This combination treatment also blocks basophil activation more efficiently than either agent alone, providing a novel approach to probe regulatory mechanisms underlying IgE hypersensitivity with implications for therapeutic interventions.

## Abstract (web summary)

Omalizumab is an antibody against IgE that is used to treat patients with asthma and chronic idiopathic urticaria. Here, the authors report the structure of omalizumab in complex with an IgE fragment and develop an approach to exchange rather than deplete IgE on human basophils to block their activation.

## Graphical abstract

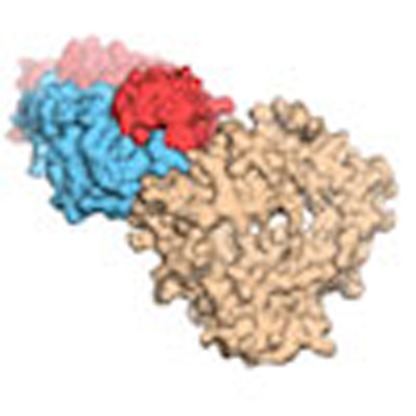

Allergic diseases represent an over-reaction of the immune system to normally non-hazardous environmental substances, such as dust mites, pet dander, pollen or mold, and the incidence of allergies worldwide is rising^1^,^2^. IgE antibodies are central to most allergic reactions, and bind to high-affinity receptors (FcɛRI) present on mast cells and basophils, sensitizing these cells to respond to allergens. FcɛRI is expressed as a trimer with one α-chain and two γ-chains or as a tetramer with an additional β-chain^3^. The FcɛRI α-chain (FcɛRIα) binds IgE with subnanomolar affinity^4^,^5^, and cells expressing FcɛRI are preloaded with IgE and primed for activation. A second IgE receptor (CD23) is expressed on additional cells, including B lymphocytes, where it is thought to play a role in IgE-mediated antigen presentation and the feedback regulation of IgE antibody production^6^,^7^,^8^,^9^,^10^,^11^,^12^.

The IgE-Fc region contains three domains (Cɛ2–4). All three domains affect IgE receptor binding, yet the Cɛ3–4 fragment independently binds FcɛRI and CD23 and contains the residues involved in IgE receptor interactions^13^,^14^,^15^ (Fig. 1a). The homodimeric IgE-Fc binds FcɛRIα asymmetrically, forming two non-equivalent contacts with FcɛRIα at the top of each Cɛ3 domain termed site 1 and site 2 (Fig. 1b) (ref. 5). Conformational rearrangements between open and closed states within the Cɛ3–4 fragment, stabilized by FcɛRIα and CD23 binding, respectively, allow FcɛRI and CD23 to function as reciprocal allosteric inhibitors (Fig. 1b) (refs 14, 15). In contrast, Cɛ2 domains do not make extensive contact with IgE receptors, but form intramolecular contacts within IgE, stabilizing a ‘bent,' asymmetric conformation of the full-length molecule (Fig. 1c). The primary role of the Cɛ2 domain is to contribute to the slow dissociation of IgE from FcɛRIα (ref. 13).

IgE has been a target for therapeutic development because of its central role in the allergic response. The anti-IgE monoclonal antibody omalizumab is currently indicated for the treatment of moderate to severe persistent asthma and chronic idiopathic urticaria. Omalizumab has demonstrated robust clinical efficacy^16^,^17^,^18^, and has promise for a wide range of other allergic conditions, including oral food allergen desensitization regimens^19^. Omalizumab acts primarily by neutralizing free serum IgE. Thereby, it also reduces surface levels of IgE on FcɛRI-expressing cells, including mast cells and basophils^20^,^21^. As IgE surface levels decline on these allergic effector cells, they lose the ability to bind allergen and to undergo IgE-dependent activation.

Omalizumab received FDA approval over a decade ago; yet, no structure of the omalizumab:IgE complex has been determined. To understand the structural basis of omalizumab:IgE interactions and its ability to inhibit both FcɛRI and CD23 binding, we determined the structure of the omalizumab Fab bound to a disulfide bond mutant of the IgE-Fc Cɛ3-Cɛ4 fragment (IgE-G335C-Fc_3–4_) to 2.5 Å (Table 1). Omalizumab binds to the IgE Cɛ3 domains outside of the FcɛRI-binding site, similar to the anti-IgE Designed Anykyrin Repeat Protein (DARPin) E2_79, in good agreement with prior mapping studies of the epitope^22^,^23^,^24^. The complex structure clarifies how omalizumab blocks IgE interactions with both the high- and low-affinity receptors. Despite the similarity in omalizumab and E2_79-binding sites on IgE, E2_79 is a disruptive inhibitor that can accelerate the dissociation of IgE:FcɛRI complexes, while omalizumab has only poor (∼1,000-fold weaker) disruptive activity^23^,^25^. Comparison of the omalizumab and E2_79 IgE complexes provides insights into the mechanism of disruptive inhibition, which could help in the development of anti-IgE antibodies with improved disruptive capabilities.

The omalizumab structure also facilitated the design of an IgE-Fc_3–4_ mutant (IgE-R419N-Fc_3–4_) that is resistant to omalizumab neutralization but is able to bind CD23 and FcɛRI. Significant experimental evidence has accumulated suggesting that IgE-dependent homeostatic regulatory pathways respond to the loss of receptor-bound IgE induced by omalizumab treatment, and could offset or constrain the therapeutic benefit of the anti-IgE treatment^8^,^12^,^26^,^27^,^28^,^29^,^30^,^31^,^32^. We demonstrate that the IgE-R419N-Fc_3–4_ mutant, in combination with omalizumab, can effectively exchange cell-bound IgE with IgE-R419N-Fc_3–4_ and that this dual inhibitor treatment is more potent at blocking basophil activation than either inhibitor alone. This approach of simultaneously depleting antigen-specific IgE, while engaging FcɛRI and CD23 receptors with an IgE variant, can be used to further probe the role of IgE-dependent regulatory pathways during anti-IgE treatment and may provide a route to enhance current anti-IgE therapies.

## Results

### Structure of the IgE–omalizumab complex

**Figure 1:**
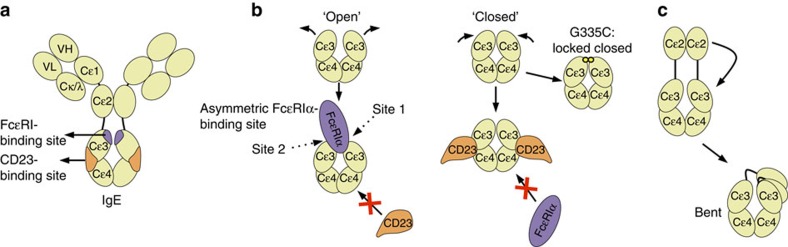
Organization and conformational rearrangements of the IgE-Fc. (**a**) IgE and the relative locations of the FcɛRIα- (purple) and CD23 (orange)-binding sites. (**b**) A representation of open and closed conformations of the IgE-Fc_3–4_ domains (including the IgE–G335C–Fc_3–4_ mutant locked in a closed conformation), and a representation of the reciprocal allosteric inhibition by FcɛRIα (purple) and CD23 (orange). (**c**) A schematic of the bent conformation of IgE, and the relative position of the Cɛ2 domains.

We previously described an IgE-Fc_3–4_ mutant (IgE-G335C-Fc_3–4_), which contains an engineered disulfide bond at position 335 that traps the IgE-Fc_3–4_ domain in a closed conformation with reduced conformational flexibility^33^ (Fig. 1b). This variant retains high-affinity binding to omalizumab, but not FcɛRIα (Supplementary Figs 1 and 2). Employing this restrained IgE-Fc_3–4_ variant, we crystallized the IgE-G335C-Fc_3–4_:omalizumab–Fab complex (subsequently referred to as the IgE:omalizumab complex). The purified complex (Supplementary Fig. 3a,b) crystalized in the P2_1_ space group, diffracted X-rays to 2.5 Å (Table 1), and the structure was solved by molecular replacement using the existing IgE–G335C–Fc_3–4_ (ref. 33) and omalizumab–Fab structures^34^. The asymmetric unit contains two copies of IgE-G335C-Fc_3–4_, and four copies of the omalizumab–Fab, providing four copies of the IgE:omalizumab interface related by non-crystallographic symmetry (NCS). Side-chain electron density was well resolved throughout the IgE:omalizumab interface (Supplementary Fig. 3c).

**Figure 2:**
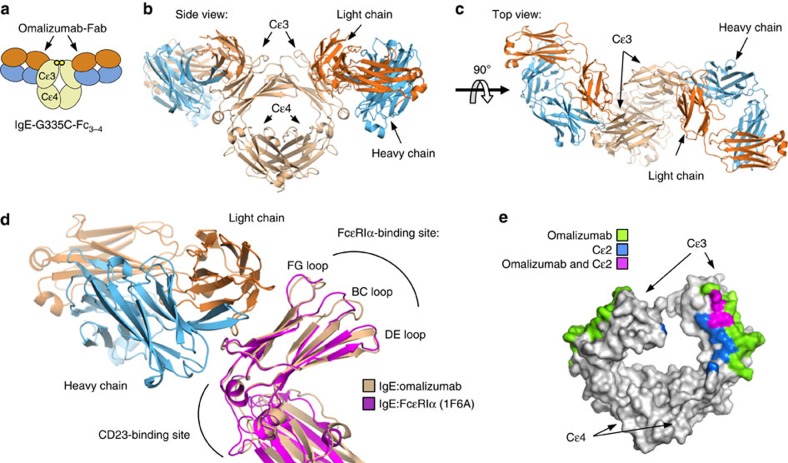
The IgE:omalizumab complex. An overview of the IgE:omalizumab complex. Blue, omalizumab heavy chain; orange, omalizumab light chain; and tan, IgE-G335C-Fc. (**a**) A cartoon diagram of the biological unit, showing IgE-G335C-Fc_3–4_ and two omalizumab Fabs binding symmetric sites. (**b**) The side view of the biologic unit of the IgE:omalizumab complex shows the omalizumab–Fab approaching perpendicularly relative to the Cɛ3–Cɛ4 domains. (**c**) The top view of the complex reveals the two nonoverlapping and symmetric omalizumab epitopes on each Cɛ3 domain within the IgE. (**d**) Comparison of IgE:omalizumab, FcɛRIα (1F6A) and CD23 (4EZM) complexes demonstrates that omalizumab binds between CD23 and FcɛRIα-binding sites within the IgE–Cɛ3 domain. Alignment of the IgE:omalizumab complex with the Cɛ3 domain of the IgE:FcɛRIα complex, in magenta, reveals no major perturbations in the FcɛRIα-binding loops. (**e**) Omalizumab and Cɛ2 directly compete for IgE-binding sites of the surface of IgE.

Analysis of the structure revealed that the omalizumab–Fab approaches perpendicularly to the IgE Cɛ3 domain, in contrast to recently proposed models^35^, and contacts symmetric binding sites on the face of the two Cɛ3 domains of the IgE dimer (Fig. 2a–c). These contacts are predominantly formed between the omalizumab–Fab and Cɛ3 β-sheet residues below the FcɛRIα-binding loops (FG, BC and DE; Fig. 2d). Structural alignment of the IgE:FcɛRIα complex^5^ with the IgE:omalizumab complex shows that the binding loops are only slightly perturbed within the IgE:omalizumab complex at receptor site 2 (Fig. 2d); thus, omalizumab does not appear to inhibit IgE:FcɛRIα interactions by distorting the FcɛRIα-binding site. Instead, the omalizumab–Fab is positioned between the binding sites of both FcɛRI and CD23, blocking interactions with both receptors, with the heavy chain proximal to the CD23 site, and the light chain proximal to the FcɛRIα-binding sites (Fig. 2d). Of note, both the position of the omalizumab epitope on the face of Cɛ3, and the perpendicular binding orientation of the omalizumab Fab, suggest that omalizumab could approach and target a preformed IgE–FcɛRI complex.

### Impact of IgE-Fc conformation on omalizumab binding

We previously demonstrated that omalizumab can bind preformed IgE-Fc_3–4_:FcɛRIα complexes, but not full-length IgE:FcɛRIα complexes^25^. Therefore, we hypothesized that the Cɛ2 domains of full-length IgE in the IgE:FcɛRIα complex obscure an omalizumab-binding site that is exposed in the IgE-Fc_3–4_:FcɛRIα complex. As predicted, alignment of the structures of IgE:omalizumab and IgE-Fc_2-4_:FcɛRIα complexes demonstrates that the Cɛ2 domains overlap extensively with an otherwise exposed omalizumab-binding epitope in the IgE-Fc_3–4_:FcɛRIα structure (Fig. 2e). Therefore, omalizumab's specificity for free IgE is not solely determined by FcɛRIα competition, but also by IgE-conformation-dependent masking of its own second omalizumab epitope. These observations suggest that fragmented IgE molecules lacking Cɛ2 could be aggregated by omalizumab, leading to basophil activation (Supplementary Fig. 4); however, we have not observed this in human basophils or mast cells^25^.

Given that the crystal structure lacks the Cɛ2 domain, it cannot account for possible contributions of the Cɛ2 to omalizumab:IgE interactions. Therefore, we also compared the binding kinetics of full-length IgE (clone Sus11) to that of the IgE-Fc_3–4_ fragments. We hypothesized that the *k*_a_ of full-length IgE might be slower than that of IgE-Fc_3–4_ because the Cɛ2 domains obscure one of the two symmetric omalizumab epitopes (Fig. 1), and, as expected, the *k*_a_ of full-length IgE was 15–30-fold lower as compared with the IgE-Fc_3–4_ fragments tested (Supplementary Fig. 1). Surprisingly, the *k*_d_ of the full-length IgE was also 4.5–10.9-fold slower than any of the three IgE-Fc_3–4_ fragments tested (Supplementary Fig. 1). This results in only minor differences in the overall equilibrium *K*_d_ (∼3.5-fold) between intact IgE and the IgE-Fc_3–4_ fragments.

The IgE Cɛ3 domains also show significant conformational flexibility, adopting closed and open states relative to the Cɛ4 domains that are associated with CD23 and FcɛRI binding, respectively. The potential impact of these conformational changes on omalizumab binding has not been fully assessed. The localization of the omalizumab epitope on the face of the Cɛ3 domain suggested that omalizumab would interact equally well with both open and closed forms of the IgE-Fc. To examine this possibility, we compared the binding kinetics of omalizumab with wild-type IgE-Fc_3–4_, the IgE-G335C-Fc_3–4_ mutant (locked in the closed conformational state^33^) and with IgE-Fc_3–4_ bound to FcɛRIα (stabilized in the open state by receptor binding). IgE-G335C-Fc_3–4_ exhibited similar kinetics in omalizumab-binding studies as wild-type IgE-Fc_3–4_ (Supplementary Fig. 1) but was unable to bind FcɛRIα (Supplementary Fig. 2). These data suggest that omalizumab can bind closed conformations of IgE efficiently, and indicate that the crystal structure reflects the normal binding mode of omalizumab for IgE-Fc_3–4._

No mutations have been identified that stabilize the open IgE-Fc_3–4_ conformational state, making studies of the omalizumab interaction with this state more challenging. However, omalizumab binds to IgE-Fc_3–4_:FcɛRIα complexes^25^, and our structural analysis demonstrates that one of the two Cɛ3 domain epitopes is fully accessible to omalizumab. Therefore, to examine the potential impact of the open Cɛ3 domain conformation on omalizumab binding, we measured the kinetics of omalizumab binding to preformed IgE-Fc_3–4_:FcɛRIα complexes. This analysis revealed an association rate constant (*k*_a_) for omalizumab with IgE-Fc_3–4_ bound to FcɛRIα complexes that was closer to full-length IgE alone and slower than unbound IgE-Fc_3–4_. The similarity in association rates between full-length IgE and IgE-Fc_3–4_:FcɛRIα complexes may be in part because of the fact that each binding partner contains a single exposed Cɛ3 domain (Supplementary Fig. 5). The dissociation rate constant (*k*_d_) for the omalizumab:IgE-Fc_3–4_:FcɛRIα complexes was also similar to that of the measured rates for IgE:FcɛRIα or omalizumab:IgE complexes (Supplementary Figs 1 and 2), consistent with the dissociation of either of these two interfaces during measurement.

Together, these kinetic data demonstrate that the IgE-Fc Cɛ3 conformations have little or no impact on omalizumab binding and that, in free IgE, the Cɛ2 domain may alter the kinetics of omalizumab binding with a small effect on the affinity of the interaction. These data support the structural observation that omalizumab:IgE interactions are primarily mediated by a stable epitope contained in the Cɛ3 domain. These kinetic data also highlight the critical role of the Cɛ2 domains in the intact receptor-bound IgE, which are required to mask an omalizumab epitope that is not directly blocked by the FcɛRI itself.

### The omalizumab epitope

**Figure 3:**
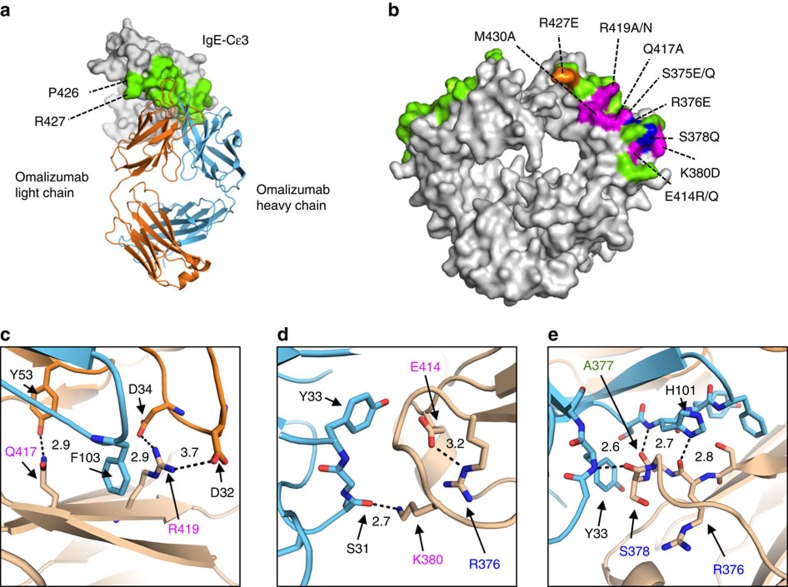
The omalizumab epitope. (**a**) A top down overview of the omalizumab:IgE complex, with interface residues coloured green and residues shared between omalizumab:IgE and IgE:FcɛRIα complexes labelled. (**b**) Position of previously published IgE heavy chain mutants and a new IgE-Fc_3–4_ mutant (R419N) at the omalizumab interface in green. Mutant residues are colour-coded by their relative binding to omalizumab (magenta 0–14%, blue 15–44% and orange 44–75% of wild-type IgE). (**c**–**e**) Detailed view of the omalizumab:IgE interface, with distances (Å) between atoms predicted to participate in hydrogen bonds or salt bridges shown in black. (**c**) A hydrogen bond between IgE Q417 and omalizumab light chain Y53, and a salt bridge between IgE R419 and light chain D34 and D32. (**d**) A hydrogen bond between IgE K380 and omalizumab heavy chain S31, and an intrachain salt bridge between IgE E414 and IgE R376, two residues implicated in omalizumab:IgE binding studies. (**e**) Hydrogen bonds observed between IgE residues R376, A377, S378 and omalizumab heavy chain residues H101 and Y33.

To establish which residues fall within the omalizumab epitope on IgE, we analysed the interfaces of the omalizumab:IgE complex with PISA^36^. All NCS copies shared the majority of contacts, which extend along the length of the Cɛ3 domain, involve 23 IgE residues and bury ∼725 Å^2^ of surface area on IgE (Fig. 3a). Previously identified IgE mutants that inhibit omalizumab binding, studied in an intact IgE heavy chain, correspond well with the binding interface observed in the crystal structure (Fig. 3b) (ref. 24). After correcting for different numbering schemes and IgE sequences from prior studies (Supplementary Fig. 6), all residues implicated in omalizumab:IgE binding are within the predicted omalizumab:IgE interface, and many participate in hydrogen bonds (R376, S378, K380, Q417) or salt bridges (R419) predicted by the crystal structure (Fig. 3c–e). Notably, IgE E414, a residue implicated in omalizumab-binding studies with the mutants E414R/Q, appears to form an intrachain salt bridge with IgE R376 (Fig. 3d) and may be essential for stabilizing the conformation of the adjacent omalizumab-binding residues (Fig. 3d,e).

The omalizumab complementarity-determining region (CDR) loops, with the exception of the light chain CDR3 loop, contact IgE. CDR residues previously shown to be required for omalizumab binding either contact IgE (light chain D32 in CDR1 (Fig. 3c) and heavy chain H101 in CDR3 (Fig. 3e)) or form interactions with neighbouring CDR loops (heavy chain CDR3 H105 and H107) (ref. 24). The complex contains five hydrogen bonds distributed throughout the omalizumab:IgE interface and two salt bridges between light chain residues D32 and D34 and IgE R419 (Fig. 3c–e). The heavy chain CDR3, which shows the most extensive conformational change from the unbound omalizumab:Fab structure (PDB ID: 4X7S), also contains three aromatic side chains that contact IgE (Y102, H101, F103; Fig. 3e). Outside of the omalizumab heavy chain CDR3, four additional aromatic side chains contact IgE: Y33 in the heavy chain CDR1, Y36 in the light chain CDR1 and Y53 and Y57 in the light chain CDR1. Therefore, it appears that a network of hydrogen bonds, salt bridges and extensive hydrophobic interactions facilitate omalizumab:IgE interactions.

The IgE residues interacting with omalizumab CDRs are largely distinct from those that engage FcɛRIα (ref. 24). IgE residues P426 and R427 are the only minor overlapping portions of the omalizumab:IgE-Fc_3–4_ and FcɛRIα:IgE-Fc_3–4_ interfaces as calculated with the PISA analysis (Fig. 3a). Each residue is unique to site 2 of the FcɛRIα:IgE complex. The residues are adjacent to the omalizumab light chain framework region (Fig. 3a and Supplementary Fig. 7) and are peripheral to the omalizumab:IgE interface. Only one NCS-related IgE chain has well-resolved electron density for R427, while the remaining copies do not (Supplementary Fig. 7). Within this chain neither R427 nor P426 make contacts (<4 Å) directly with omalizumab; however, R427 indirectly interacts with the light chain framework through a sulfate ion (Supplementary Fig. 7). Mutations at R427 (R427E) lead to a minor reduction in omalizumab binding (25–56%), suggesting that this residue can affect omalizumab:IgE interactions^24^. In contrast, the R427E mutation substantially reduced FcɛRIα binding to IgE, while another mutant series that contained a R427A mutation only partially reduced FcɛRIα binding^37^. Although omalizumab may directly compete with FcɛRIα for IgE residues, the extent of direct competition and binding site overlap involves at most two amino acids.

### The structural basis of FcɛRI and CD23 competition

Omalizumab inhibition of FcɛRIα and CD23 binding could arise from contributions of multiple structural mechanisms, including direct competition for receptor-binding residues on IgE, steric clashes caused by physical overlap of omalizumab and IgE receptors and potential omalizumab-induced conformational changes in IgE. The kinetic and structural data suggest that omalizumab does not induce conformational changes in FcɛRIα-binding loops or in the relative positions of the Cɛ3 domains that could affect receptor binding. Omalizumab also shows minimal overlap with FcɛRIα-binding residues; however, physical overlap between the bound omalizumab Fab and FcɛRIα could be substantial and critical to omalizumab activity.

**Figure 4:**
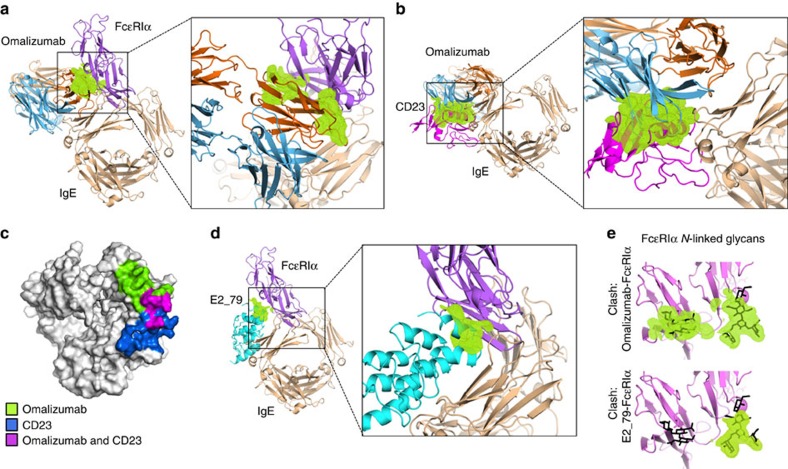
The structural basis of omalizumab FcɛRIα and CD23 competition. (**a**) Structural alignment of complexes reveals that atomic overlap (green) between the omalizumab light chain (orange) and FcɛRIα (purple) would allow omalizumab to block IgE binding at site 2. (**b**) Steric conflicts (green) between the omalizumab–Fab heavy chain (blue) and CD23 (magenta) as well as direct competition for binding sites (**c**) appear to drive omalizumab inhibition of CD23:IgE interactions. (**d**) The disruptive DARPin inhibitor E2_79 (cyan) has a similar binding mode to the omalizumab Fab, yet has less atomic overlap with FcɛRIα in aligned structures of the complexes. (**e**) The majority of steric clashes (green) between omalizumab and FcɛRIα and E2_79 and FcɛRIα occur with *N*-linked glycans (black) found on FcɛRIα.

To gain quantitative insight into the contribution of inhibitor overlap in blocking IgE interactions with FcɛRIα and CD23, we calculated the theoretical volumes of atomic overlap between omalizumab and its two IgE receptors. First, we performed a structural alignment of the Cɛ3 domain of the IgE:omalizumab complex with the Cɛ3 domain of the IgE:FcɛRIα complex at sites 1 and 2 (Fig. 4a). This alignment strategy accounts for the variability of open and closed IgE conformations observed across IgE crystal structures^14^,^33^,^38^. We then calculated the volume of atomic overlap between the superimposed omalizumab and FcɛRIα proteins. This analysis revealed that, for the omalizumab-binding site proximal to FcɛRIα binding site 2, there are significant steric clashes between the antibody light chain and both domains of the FcɛRIα receptor (Fig. 4a), while no clashes exist at site 1. These structural data indicate that omalizumab's mechanism of FcɛRIα inhibition involves substantial steric conflict with the receptor at site 2, while direct competition for FcɛRIα-binding residues is limited.

Omalizumab has also been shown to inhibit the binding of CD23 (ref. 39). Both substantial steric overlap between omalizumab and CD23, and direct competition for IgE-binding residues by the omalizumab heavy chain, contribute to omalizumab inhibition of CD23 binding (Fig. 4b,c). The degree of steric overlap of omalizumab with CD23 is significantly greater than that observed for FcɛRIα (Fig. 4b). Binding-site comparisons also demonstrate a more extensive overlap between IgE residues that engage omalizumab and CD23 in their respective complexes as compared with FcɛRIα (Fig. 4c).

### Steric overlap and inhibitor-induced FcɛRIα complex dissociation

We recently described a class of IgE inhibitors derived from Designed Ankyrin Repeat Protein (DARPin) libraries, capable of disrupting IgE:FcɛRI complexes^23^,^25^. These agents target preformed IgE:FcɛRIα complexes found on mast cells and basophils and accelerate the dissociation rate constant to release free IgE. The activated release of IgE on the surface of effector cells might prove beneficial in treating acute allergic reactions and enhance the clearance of allergen-specific IgE during anti-IgE therapy by targeting both cellular and serum pools of IgE simultaneously. We have demonstrated that a bivalent DARPin (bi53_79) containing a non-competitive IgE-binding domain and a disruptive competitor domain dissociates complexes with greater efficiency *in vitro* and shows greater potency than omalizumab in blocking passive cutaneous anaphylaxis in mice bearing the human FcɛRI receptor^25^. To our surprise, we also observed that omalizumab is not strictly a competitive inhibitor of IgE:FcɛRIα interactions, but at higher concentrations it is also capable of targeting and disrupting IgE:FcɛRIα complexes^25^. We have published the crystal structure of the DARPin-based inhibitor E2_79 (ref. 23), which is able to accelerate the dissociation of FcɛRIα complexes at concentrations ∼3,000 × above the E2_79:IgE K_D_ (ref. 23). In contrast, omalizumab shows an ability to disrupt preformed FcɛRIα complexes at concentrations that are much higher (∼1,000,000-fold greater) than the omalizumab:IgE K_D_ (ref. 25), indicating that it is much less efficient at the process of binding to and dissociating these preformed complexes. We hypothesized that this difference in disruptive capability was related to the binding-site locations for E2_79 and omalizumab on IgE and the level of atomic overlap between each inhibitor and receptor. We therefore compared the structure of both the E2_79:IgE-G335C-Fc_3–4_ complex (E2_79:IgE) and the omalizumab:IgE complex.

The structure of the E2_79:IgE complex demonstrated that the E2_79-binding sites do not overlap with FcɛRIα-binding residues, while omalizumab exhibits only minor peripheral interactions with two FcɛRIα-binding residues^23^. Instead, similar to omalizumab, E2_79 exhibited steric conflicts with FcɛRIα in aligned structures of the complexes. Given that both agents can disrupt preformed IgE:FcɛRIα complexes^25^, we sought to quantitatively compare their relevant steric clashes with FcɛRIα when bound to IgE, by computing the predicted volume of atom–atom overlap of each inhibitor with FcɛRIα in aligned complex structures. This analysis reveals that omalizumab has roughly three times the volume of atomic overlap with FcɛRIα compared with E2_79 (omalizumab and FcɛRIα=1,183 Å^3^ versus E2_79 and FcɛRIα=401 Å^3^). The omalizumab steric conflicts extend along the length of the omalizumab light chain and FcɛRIα *N*-terminal domain, while the E2_79 steric conflicts are more localized near the IgE:FcɛRIα interface (Fig. 4a-4d) (ref. 40). Since the E2_79 and omalizumab-binding sites are substantially overlapping on the IgE-Fc (Supplementary Fig. 8), this large difference in steric overlap with FcɛRIα stands out as a prominent structural feature that correlates with the relative disruptive activities of these inhibitors. Conformational dynamics in the IgE:FcɛRIα complex may transiently allow E2_79 association and subsequent acceleration of FcɛRIα dissociation^23^. Given the significantly larger region of steric conflicts observed between omalizumab and FcɛRIα, conformational states of the IgE:FcɛRIα that could accommodate omalizumab association may simply be less accessible and/or occur with lower frequency, explaining its lower activity. A significant fraction of the steric clashes with both inhibitors occur between the protein backbone of omalizumab or E2_79 and carbohydrate groups on FcɛRIα (Fig. 4e). These carbohydrate groups likely explore a wider range of conformations, and may help facilitate the association of these inhibitors to preformed IgE:FcɛRIα complexes.

### A single IgE mutation prevents omalizumab binding

**Figure 5:**
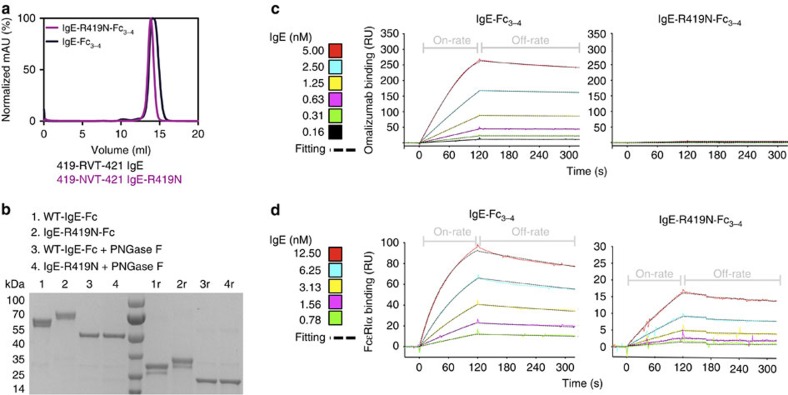
A single IgE mutation prevents omalizumab binding. (**a**) The IgE-Fc_3–4_ point mutant, R419N, contains a novel glycosylation consensus sequence and is expressed as a soluble monomer as assessed by gel filtration chromatography of IgE-R419N-Fc_3–4_ and IgE-Fc_3–4_ species. (**b**) SDS–PAGE analysis of non-reduced and reduced (‘r') IgE-R419N-Fc_3–4_, and IgE-Fc_3–4_, demonstrates that the R419N mutation induces an additional glycosylation event and a mass shift of ∼2 kDa per IgE chain. PNGaseF treatment removes all *N*-linked glycans, and demonstrates that the mass shift in the IgE-R419N-Fc_3–4_ protein arises from *N*-linked glycosylation. (**c**,**d**) SPR-binding assays with immobilized omalizumab (**c**) or FcɛRIα (**d**) show that IgE-R419N-Fc_3–4_ is unable to bind omalizumab, but exhibits binding to FcɛRIα at nM concentrations.

To further validate observations from the crystal structure, we produced an additional IgE-Fc_3–4_ mutant. IgE residue R419 lies at the interface of the IgE:omalizumab complex, forming contacts with both light and heavy chain CDR loops (Fig. 3c) and participating in salt bridges. Mutation of R419 to an asparagine (R419N) introduces the glycosylation consensus sequence—asparagine valine threonine (NVT) (Fig. 5a). We hypothesized that by mutating this residue we would abolish omalizumab binding by introducing a glycosylation site at the core of the omalizumab epitope.

The R419N mutation induced a shift in the mass of recombinant IgE-Fc_3–4_ as assessed by SDS–PAGE and gel filtration, consistent with the introduction of an additional *N*-linked glycan (Fig. 5a,b). The IgE-Fc_3–4_ contains two *N*-linked glycosylation sites, N371 and N394 (refs 41, 42). We have found in recombinant preparations of IgE-Fc_3–4_ that glycosylation at N371 is heterogeneous and leads to a minor band in purified material (Fig. 5b). Both major and minor species of IgE-R419N-Fc_3–4_ remain in similar proportions to wild-type IgE-Fc_3–4_ species, with similar mobility shifts in SDS–PAGE. Furthermore, the shift in apparent mass for IgE-R419N-Fc_3–4_ relative to wild-type IgE-Fc_3–4_ was lost on digestion with PNGaseF, confirming that it is caused by *N*-linked glycosylation (Fig. 5b). This additional glycosylation in the middle of the omalizumab epitope completely abolishes omalizumab binding, but only slightly perturbs IgE-R419N-Fc_3–4_ binding to FcɛRIα as assessed by surface plasmon resonance (SPR; Fig. 5c,d and summary data Supplementary Figs 1 and 2). Taken together, these data demonstrate that the R419N mutation introduces a novel glycosylation site to yield an omalizumab-resistant IgE-Fc_3–4_ variant.

### Exchange of IgE on human basophils

The paradigm of using anti-IgE treatment to deplete free IgE (both allergic and non-allergic species) has proven successful for controlling allergic diseases. During omalizumab therapy, omalizumab neutralizes free serum IgE and slowly decreases surface levels of IgE on allergic effector cells. Therefore, it is impossible to simultaneously replace depleted allergen-reactive species with benign IgE species because of the requirement for continued excess omalizumab to be present during treatment. Since IgE-dependent homeostatic regulatory pathways could potentially counteract anti-IgE therapies, we sought to explore the possibility of replacing rather than removing patient IgE. We hypothesized that omalizumab-resistant IgE variants or fragments could be used in combination with omalizumab to effectively exchange the native IgE on human cells bearing FcɛRI or CD23.

Over the course of omalizumab treatment, free IgE levels and IgE surface levels on basophils decline relatively rapidly within days^43^, while mast cell IgE levels persist for significantly longer^44^. The rapid decline of basophil-associated IgE may in part be driven kinetically by basophil turnover *in vivo*^45^. However, within the experimental timeframe for our *ex vivo* experiments with human whole blood (24–48 h), we did not observe significant loss of cell surface IgE at doses corresponding to the mean omalizumab serum concentrations from clinical trials^46^. Therefore, we employed supraphysiologic doses of omalizumab (25 μM) to enhance the removal of IgE as described previously^25^. These doses were used here to accelerate the loss of cell-bound IgE to facilitate these exchange experiments. Nonetheless, lower therapeutic doses of omalizumab are sufficient to capture free IgE and reduce cell surface levels of IgE over time, which is the basis of omalizumab's therapeutic effect.

To track the addition, removal and exchange of IgE species, we employed biotinylated IgE-Fc_3–4_ or omalizumab-resistant IgE-R419N-Fc_3–4_, and the JW8 human/mouse chimeric IgE (composed of a human IgE heavy chain and a mouse-λ-light chain). The JW8 mouse-λ-light chain allowed us to track IgE reloading on stripped basophils in parallel with the biotinylated IgE-Fc_3–4_ proteins. We ensured that all antibody reagents used in staining carried the mouse-κ-light chain, or were rat antibodies, to avoid nonspecific binding from the anti-mouse-λ-light-chain antibody used to track JW8-IgE.

**Figure 6:**
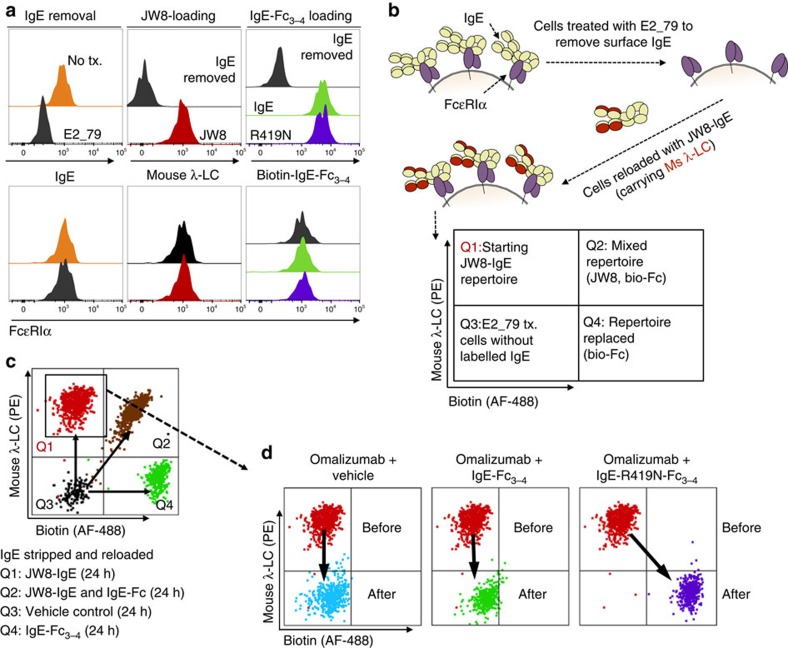
Exchange of IgE on human basophils. (**a**) Left panel: 2-h treatment with 25 μM E2_79 removes surface IgE from primary human basophils, but does not alter surface FcɛRIα levels within 24 h. Middle panel: E2_79-treated basophils can be reloaded with JW8-IgE and tracked by JW8's mouse-λ-light chain (λ-LC). Right panel: biotinylated WT and IgE-R419N-Fc_3–4_ variants bind E2_79-treated basophils. (**b**) Experimental design for the IgE exchange. In brief, IgE is removed from primary basophils using E2_79. JW8-IgE is then reloaded on basophils to generate a traceable starting IgE population. (**c**) Experimental validation shows distinct populations of basophils with JW8-IgE (Q1), JW8-IgE and biotinylated IgE-Fc_3–4_ (Q2), biotinylated IgE-Fc_3–4_ alone (Q4) and E2_79-treated cells without labelled IgE (Q3; displaying merged dot plots from each sample). (**d**) All panels show starting JW8-reloaded population in Q1 before treatment, and cells after treatment. Left panel: overnight treatment with high-concentration omalizumab (25 μM) is sufficient to remove the majority of JW8-IgE. Middle panel: overnight treatment of cells with omalizumab (25 μM) and IgE-Fc_3–4_ (1 μg ml^−1^ or ∼18 nM) results in depletion of JW8-IgE, but no exchange for IgE-Fc_3–4_. Right panel: overnight treatment of cells with omalizumab (25 μM) and IgE-R419N-Fc_3–4_ (1 μg ml^−1^ or ∼18 nM) results in depletion of JW8-IgE, and IgE exchange to IgE-R419N-Fc_3–4_. (Representative dot plots shown. *N*=3 at 10 μg ml^−1^ IgE-Fc doses and controls, and *N*=3 at 1 μg ml^−1^ IgE-Fc doses).

We isolated blood from three donors, and depleted surface IgE on basophils by treating each sample with the disruptive inhibitor E2_79 (Fig. 6a, gating scheme Supplementary Fig. 9). We then reloaded the cells with JW8-IgE (Fig. 6a), while also verifying that stripped cells could be reloaded with biotinylated IgE-Fc_3–4_ or IgE-R419N-Fc_3–4_ species (Fig. 6a). Therefore, we could remove native IgE species, reload cells with homogeneous traceable IgE and subsequently track the removal or exchange of JW8-IgE (Fig. 6b,c). The λ-light-chain-specific staining of JW8 and biotin-specific staining of the IgE-Fc_3–4_ variants distinguished human basophils that had no JW8-IgE or biotinylated-IgE-Fc_3–4_, a mix of both species, or a single IgE species (Fig. 6b,c).

Overnight omalizumab treatment of cells that had been homogenously reloaded with JW8-IgE completely removed the JW8-IgE from the cell surface, as shown by an overlay of the pre- and post-treatment flow cytometry profiles (arrow, Fig. 6d). Co-administration of IgE-Fc_3–4_ and omalizumab also showed complete depletion of JW8-IgE species but no exchange for IgE-Fc_3–4_ at 1 μg ml^−1^ IgE doses (Fig. 6d). In contrast, co-administration of omalizumab-resistant IgE-R419N-Fc_3–4_ with omalizumab depleted JW8-IgE surface levels, and facilitated IgE-R419N-Fc_3–4_ reloading to levels observed in IgE-stripped basophils treated with IgE-R419N-Fc_3–4_ alone (Fig. 6d). Therefore, co-administration of omalizumab and IgE-R419N-Fc_3–4_ effectively exchanges the receptor-bound IgE on human basophils for IgE-R419N-Fc_3–4_ *ex vivo*. Even when wild-type IgE-Fc_3–4_ doses far beyond physiologic IgE levels were employed (10 μg ml^−1^), only minimal reloading of wild-type IgE-Fc_3–4_ was observed, in contrast to the dramatic reloading of IgE-R419N-Fc_3–4_ (Supplementary Fig. 10). This effect was not restricted to FcɛRI-expressing basophils. CD23^+^ B-cells treated with omalizumab and IgE-Fc_3–4_ variants retained surface IgE-R419N-Fc_3–4_, but were stripped of wild-type IgE-Fc_3–4_ (Supplementary Fig. 11b). Furthermore, both wild-type IgE-Fc_3–4_ and IgE-R419N-Fc_3–4_ occupancy of the CD23 receptor stabilized surface CD23 and increased CD23 surface levels (Supplementary Fig. 11c). Importantly, IgE-mediated stabilization of surface CD23 prevents the release of soluble CD23 (sCD23), a soluble mediator shown to upregulate IgE expression^8^,^32^.

### IgE-R419N-Fc_3–4_ and omalizumab act synergistically

**Figure 7:**
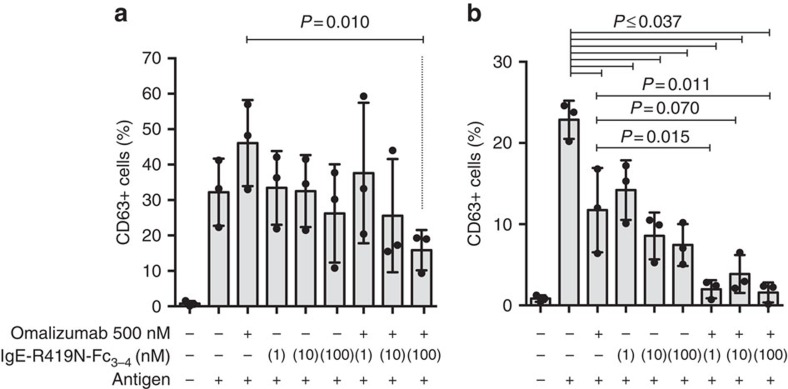
IgE-R419N-Fc_3-4_ and omalizumab act synergistically. Human basophils from three healthy volunteers were isolated, stripped of native IgE by bi53_79 DARPin treatment and reloaded with NP-reactive JW8-IgE. These NP-reactive basophils were then cultured with or without omalizumab and IgE-R419N-Fc_3-4_ as indicated for 3 (**a**) or 6 (**b**) days before antigen challenge. Basophil activation was assessed by the per cent of CD63-positive cells. There was a statistically significant difference between groups as determined by a repeated-measures analysis of variance (*F*=7.4, *P*=0.0004 Day 3 and *F*=19.3, *P*=0.0001 Day 6), and a Tukey *post hoc* test was used to determine the significance of differences between groups. On day 6, the untreated basophils showed reduced activation, which likely reflects the spontaneous loss of IgE.

To determine whether IgE-R419N-Fc_3–4_ could enhance the effect of omalizumab treatment in human cells, we performed basophil-activation tests. Basophils were isolated from healthy donors, treated with DARPins to remove surface IgE (using the enhanced bi53_79 DARPin^25^) and reloaded with the 4-hydroxy-3-nitrophenylacetyl (NP)-specific JW8-IgE. The resultant NP-reactive basophils were then left untreated or were treated with omalizumab (at therapeutic concentrations of 500 nM), IgE-R419N-Fc_3–4_ or combinations of both agents for 3 (Fig. 7a) or 6 days (Fig. 7b) before NP-antigen challenge. Given that IgE:FcɛRIα complexes dissociate slowly, and omalizumab is a weak disruptive inhibitor at the therapeutically relevant concentration used, we did not anticipate a rapid response in basophils homogenously reloaded with JW8-IgE. Accordingly, after 3 days, there were no significant reductions in basophil responses as compared with untreated controls (Fig. 7a). Of note, omalizumab transiently increased basophil sensitivity on day 3, although this effect was overcome by day 6, presumably as omalizumab neutralized a greater fraction of JW8-IgE previously bound to cells. Although this effect was not significant, it does fit with clinical studies, which suggest that basophil sensitivity is increased on a per IgE basis during omalizumab therapy^30^. There was significantly less activation in cells treated with a combination of omalizumab and IgE-R419N-Fc_3–4_ (100 nM) as compared with omalizumab alone by day 3 (Fig. 7a), suggesting that combination therapy could offset this transient effect in omalizumab-treated samples. By day 6, a significant reduction in basophil activation was observed in all treatment arms as compared with the untreated controls (Fig. 7b). This result was surprising, given that IgE-R419N-Fc_3–4_ alone was able to inhibit basophil reactivity at 1 nM concentrations (Fig. 7b). Furthermore, in combination with omalizumab, 1 nM IgE-R419N-Fc_3–4_ almost completely inhibited basophil activation (Fig. 7b). The additive effect of omalizumab (500 nM) and IgE-R419N-Fc_3–4_ (1 nM) should be minor if each agent acted only as a competitive inhibitor for JW8-IgE:FcɛRIα interactions, suggesting that these agents can act synergistically to block basophil activation.

## Discussion

Anti-IgE therapy remains an important tool for the management of allergic disorders, and novel second-generation anti-IgE therapies will soon join omalizumab. Yet, the aetiology of allergic disorders is complex and optimal therapeutic responses will likely come from treatment regimens that target and modulate multiple immunological processes.

Here we have clarified the structural basis for omalizumab-mediated inhibition of IgE binding to both FcɛRI and CD23. This analysis revealed similarities between the disruptive inhibitor E2_79 and omalizumab, and provides a mechanistic framework to develop antibody-based therapies that can accelerate the dissociation of IgE:FcɛRIα complexes. Such agents could rapidly disarm basophils and mast cells, allowing them to achieve a therapeutic effect faster.

We have also demonstrated that mutant IgE fragments, in conjunction with omalizumab treatment, can exchange native IgE on human cells for IgE fragments, maintaining the occupancy of both high- and low-affinity IgE receptors. We pursued this approach, given the experimental evidence that basophils increase in sensitivity on a per IgE basis during omalizumab treatment^28^,^29^,^30^, and that IgE production can be regulated by IgE:CD23 signalling^8^,^11^,^12^,^31^,^32^. These observations raise significant questions about the impact of therapeutic IgE depletion, and suggest that homeostatic responses to the loss of IgE could offset or constrain the therapeutic benefit of anti-IgE treatment. Our experimental observations provide a system to test some of these regulatory pathways and could potentially be used as an adjunct therapy with omalizumab. To this end, we have also demonstrated that IgE-R419N-Fc_3–4_ can act synergistically with omalizumab *ex vivo* at very low doses to inhibit basophil activation. This finding suggests that simultaneously targeting FcɛRI and IgE with competitive inhibitors could enhance therapeutic responses. Prior studies have demonstrated that non-activating ligands can antagonize FcɛRI responses to activating ligands by sequestering receptor-proximal signalling components^47^, and it is possible that IgE-R419N-Fc_3–4:_FcɛRI complexes could suppress activation through such mechanisms, in addition to competing for receptor occupancy. Sustained FcɛRI receptor occupancy could also suppress homeostatic responses to the loss of IgE on omalizumab-treated basophils.

Finally, the dissociation of allergen-specific IgE on mast cells located in peripheral tissues is slow, and the effectiveness of anti-IgE within these compartments is poorly understood^44^. Given that the FcɛRI on mast cells has been shown to drive IgE tissue localization^48^, co-administration of omalizumab and omalizumab-resistant IgE fragments that bind FcɛRI may enhance the exchange of allergen-specific IgE in peripheral sites, and contribute to the therapeutic benefit of anti-IgE treatment.

Despite the potential utility of IgE exchange, and promising *ex vivo* results, the concept is far from clinical application. Unexpected antibody responses to the R419N IgE, although unlikely, could prevent the development of any universally benign IgE variant for exchange therapy, and could induce anaphylactic antibody responses to receptor-bound IgE fragments. Yet, our approach with IgE-R419N-Fc_3–4_ does have two distinct advantages in this regard. First, *N*-linked glycosylation events are effective at masking antibody epitopes, and could reduce the immunogenicity of IgE-R419N-Fc_3–4_ (ref. 49). Second, the new antigenic surface generated by truncating IgE to the Cɛ3-4 domains would be largely inaccessible once bound to FcɛRIα, preventing any antibody responses to this epitope from crosslinking receptor-bound IgE fragments. Given that anti-IgE and anti-FcɛRIα antibody responses are relatively common, and are not always pathological^50^, it is possible that such an approach could be well tolerated.

## Methods

### Preparation of omalizumab and recombinant proteins

Fab fragments of Omalizumab (Novartis) were prepared by digestion over an immobilized ficin agarose resin (Pierce) in 10-mM citrate buffer with 25 mM cysteine and 5 mM EDTA at pH 6.0 for 5 h. The Fab fragments were purified in two steps with protein G (Pierce) and gel filtration to yield homogenous omalizumab–Fab. Protein G columns were washed with phosphate buffer over a pH gradient (8.0, 7.0, 6.0 and 5.0) and Fab was eluted with glycine buffer at pH 2.5. Insect-cell-derived IgE-Fc_3–4,_ IgE-G335C-Fc_3–4_ and FcɛRIα were expressed in High Five insect cells and purified using Ni-NTA affinity chromatography and further purified using gel filtration on a Superdex 200 10/300 GL column (GE). All insect vectors have been previously published: IgE-Fc_3–4_ (ref. 33), IgE-G335C-Fc_3–4_ (ref. 33) and FcɛRIα^51^. Mammalian-derived IgE-Fc_3–4_ and the R419N mutant, used in all cell-based assays, were cloned into the pYD7 vector (National Research Council (NRC), Canada) with a vascular endothelial growth factor (VEGF) signal sequence from the pTTVH8G vector (NRC). Constructs were transfected using 25-kDa linear polyethylenimine (Polysciences), and transiently expressed in suspension HEK-6E cells (NRC) for 120 h according to the supplier's protocols. Cell supernatants were filtered through 0.45-μM Durapore filter (Millipore) and incubated with Ni-NTA resin (Qiagen) for 1 h at room temperature, washed with 10 resin bed volumes of wash buffer (25 mM imidazole in PBS at pH 7.4) and eluted with 2 resin bed volumes of elution buffer (300 mM imidazole in PBS at pH 7.4). The eluted protein was then concentrated using an Amicon Ultra-15 filter unit (Millipore) and further purified with gel filtration on a Superdex 200 10/300 GL column (GE). Protein used for experiments with human basophils was subsequently buffer-exchanged into sterile PBS pH 7.4 using an Amicon Ultra-15 filter unit (Millipore). The DARPins E2_79 and bi53_79 were cloned into the pQE-30 expression vector between BamHI and HindIII restriction sites, and expressed in XL-1 blue E. coli (NEB) at 37 degrees overnight following induction with isopropyl-β-D-thiogalactoside. Soluble DARPins were purified using Ni-NTA affinity chromatography, followed by gel filtration on a Superdex 200 10/300 GL column (GE).

### Crystallization conditions

Crystals were grown in hanging drops in 0.2 M Lithium Sulfate, 0.1 M Tris pH 8.5 and 41% PEG 400. A volume of 0.1 μl of complex at 9 mg ml^−1^ was added to 0.1 μl of well solution. Crystals were harvested and frozen in the same crystallization buffer for data collection.

### Structure determination of the IgE:omalizumab complex

Diffraction data were collected in two 360° sweeps of one crystal on the microfocus beamline 12-2 at the Stanford Synchrotron Radiation Lightsource, and data were indexed, integrated and scaled using the HKL2000 suite^52^. The omalizumab-C335 crystals grew in the P2_1_ space group with the unit cell dimensions *a*=100.10 Å, *b*=107.14 Å, *c*=151.04 Å and *β*=95.18°. A molecular replacement solution was obtained using Phaser-MR in the Phenix package (version 1.9)^53^ using the models 4GT7 (for C335-IgE-Fc)^33^ and 4X7S (for omalizumab–Fab)^34^. Automated model building to improve early models was performed using Phenix AutoBuild, and refinement was performed using rounds of Phenix Refine and manual model building with Coot (version 0.8.1) (ref. 54). NCS restraints were identified automatically in phenix.refine by sequence similarity and default root mean squared deviation tolerance of <2 Å. NCS restraints were applied in early rounds of refinement and removed in final rounds of refinement. The final model was refined to 2.5 Å (Table 1) and was validated using MolProbity and Phenix comprehensive validation. The model had Ramachandran-favoured conformations in >97% of residues and 0.2% of residues were outliers. The relatively high B-factors after refinement are expected, given the high-average Wilson B-factor (61.89).

### Calculation volume overlaps

Structural alignments with omalizumab:IgE and E2_79:IgE complexes were made with the Cɛ3 domain at site 2 of the FcɛRIα complex. The alignment with the CD23:IgE complex was made with either Cɛ3 domain within the CD23 complex (similar results were found with both alignments in this symmetric complex). The coordinates of each IgE-binding molecule (FcɛRIα, omalizumab, E2_79 and CD23) from these aligned complexes were then loaded into pdbset (CCP4) and centred within an arbitrarily defined unit cell to accommodate the full chain. The coordinates of the aligned and transformed structures were then input into sfall (CCP4) to generate an atom map from the polypeptide chain (solvent atoms and ligands were not included), and input into mapmsk (CCP4) to make a map mask. The resulting map masks were input into overlapmap (CCP4) using the MAP INCLUDE function, keeping only density of overlapping regions of each inhibitor with the receptor complex in question. The volume of the resulting overlap maps were then calculated with the volumes tool in Chimera^40^.

### Biotinylation of proteins

IgE-Fc_3–4_ or R419N-IgE-Fc_3–4_ was biotinylated using EZ-Link Sulfo-NHS-LC-biotin (Pierce), with a 30-fold molar excess of sulfo-NHS-LC-biotin for 30 min at room temperature. The reaction was stopped using 1.0 M Tris pH 7.4, and proteins were dialysed overnight into PBS pH 7.4, and sterile-filtered with 0.22-μm filter.

### Basophil IgE exchange experiments

Blood was drawn from two healthy volunteers and a third volunteer with a history of food allergy ranging in age from 21 to 37 (the protocol for this study was approved by the Institutional Review Board of Stanford University, and all informed consent was obtained from all subjects). Blood was collected in heparin vacutainer tubes (BD), and washed in 10 blood volumes of BF buffer (RPMI-1640 (Life Technologies) supplemented with 10% fetal calf serum (FCS; Gibco) and 1% penicillin/streptomycin (Gibco)). Washed blood cells were then suspended in their original blood volume in BF buffer and treated with or without E2_79 at 25 μM for 2 h at 37 °C to remove surface IgE. Stripped cells were then reloaded with IgE-JW8 at 120–300 ng ml^−1^ as estimated from supplier's supplied concentration range (AbD Serotec) or were left untreated. These cells were then treated overnight at 37°, as specified in the figure legends with omalizumab, IgE-Fc variants or vehicle controls. The following day cells were washed three times with BF buffer at 4 °C and stained for analysis.

### Flow cytometry for basophil IgE exchange

After washing, treated cells and controls were incubated with human Fc-block (BD), and stained with the following antibodies as described in figure legends (at 1:100 dilution unless stated): IgE-FITC (eBiosciences clone: IgE21), FcɛRIα-APC (eBiosciences clone AER-37 at 1:50 dilution), anti-biotin AF-488 (eBiosciences clone: BK-1/39), anti-mouse-lambda-light-chain PE (BioLegend clone: RML-42), CD123 PE-Cy5 (BD clone: 9F5 at 1:20 dilution), HLA-DR PE-Cy7 (BioLegend clone L243 at 1:80 dilution), CD203c BV421 (BioLegend clone: NP4D6 at 1:50 dilution), CD19-PE (BD clone: HIB19 at 1:50), CD23-BV421 (BioLegend EBVCS-5 at 1:50 dilution) and Aqua Live Dead stain (Life Technologies). Stained cells were then lysed with RBS lysis buffer (BioLegend) for 5 min at room temperature, and washed with FACS buffer (PBS pH 7.4 supplemented with 10% FCS). Data were collected on a DxP FACSCAN from Cytek Development in Fremont, CA (10 colours with three lasers—488, 639, 407) using FlowJo CE and analysed using FlowJo (version 10).

### SPR assays

SPR measurements were conducted on a BIAcore X100 device and evaluated with the BIAevaluation software (GE Healthcare, Fairfield, USA). For kinetic analysis of different IgE fragments on omalizumab and FcɛRIα, 1,000 response units of omalizumab or rhFcɛRIα were immobilized in acetate buffer (pH 4.5 for omalizumab and pH 4.0 for rhFcɛRIα) on flow cell 2 of a CM5 chip (GE Healthcare). Flow cell 1 was activated and deactivated without immobilization, according to the manufacturer's protocol. The different IgE fragments (produced as described above) or full-length Sus11 IgE were diluted in HBS-EP+ running buffer (GE Healthcare) and injected for 120 s at a constant flow rate of 10 μl min^−1^. Dissociation was assessed for 240 s under constant buffer flow. For each measurement, the chip surface was regenerated with 50 mM NaOH. Individual sensorgram curves were exported to Excel, and graphs were prepared with the GraphPad Prism 5.0 software (GraphPad Software, La Jolla, USA). In all experiments, unspecific binding to flow cell 1 was subtracted from the signal on flow cell 2.

### Functional assay with primary human basophils

Human primary basophils were isolated from whole blood of volunteers with approval from the local ethics committee (KEK Bern). Informed consent was obtained from all donors in accordance with the Helsinki Declaration. Human basophils were isolated from three different donors, with total IgE levels ranging from 35 to 78 kU l^−1^ by using Percoll density centrifugation of dextran-sedimented supernatants with further purification with the Miltenyi basophil isolation kit II (Miltenyi Biotec, Bergisch Gladbach, Germany), as previously described^55^. Total IgE levels of the donors were determined using ImmunoCAP (Phadia, Uppsala, Sweden). Purified primary human basophils were seeded at 0.5 × 10^5^ cells per well in a 96-well plate in 50 μl of RPMI containing 10% heat-inactivated FCS, 100 IU ml^−1^ penicillin and 100 μg ml^−1^ streptomycin (medium). Cells were kept in a cell incubator at 37 °C, 5% CO_2_. For desensitization, cells were treated with 50 μM disruptive anti-IgE inhibitor bi53_79 for 8 h in the presence of 10 ng ml^−1^ rhIL-3 and subsequently washed three times with 150 μl PBS to remove dissociated IgE and anti-IgE inhibitor from the supernatant. For resensitization, cells were incubated with 100 nM JW8-IgE (NBS-C BioScience, Vienna, Austria) for 2 h in the presence of 10 ng ml^−1^ rhIL-3. Subsequently, cells were washed two times with 150 μl PBS and treated with omalizumab, IgE-R419N-Fc_3–4_ or a combination of these molecules for 3 or 6 days at the indicated concentrations. For determination of basophil activation, cells were stimulated with 1–1,000 ng ml^−1^ NIP(7)BSA (BioSearch Technologies, Petaluma, USA) in the presence of 10 ng ml^−1^ rhIL-3 for 30 min at 37 °C, 5% CO_2_. Subsequently, cells were stained with 10 μl anti-CD63 FITC anti-CCR3-PE staining mix (FK-CCR Flow CAST Bühlmann Laboratories AG, Schönenbuch, Switzerland) for 20 min at room temperature. At least 3 × 10^3^ basophils were acquired on a FACSCalibur device. Data were analysed with the FlowJo V10 software (TreeStar, Ashland, OR).

## Additional information

**Accession codes:** The reflection data and coordinates for the omalizumab:IgE complex have been deposited in the RCSB with PDB ID code 5HYS.

**How to cite this article:** Pennington, L. F. *et al*. Structural basis of omalizumab therapy and omalizumab-mediated IgE exchange. *Nat. Commun.* 7:11610 doi: 10.1038/ncomms11610 (2016).

## Supplementary Material

Supplementary Information Supplementary Figures 1-11

## Figures and Tables

**Table 1:** Data collection and refinement statistics.

	**IgE-G335C-Fc**_**3–4**_**:omalizumab–Fab**
*Data collection*	
Space group	P 1 21 1
Cell dimensions	
a,b,c (Å)	100.10, 107.14, 151.04
α,β,γ (°)	90.00, 95.18, 90.00
Resolution (Å)	37.61–2.50 (2.59–2.50)
*R*_merge_	0.115 (2.009)
CC1/2	0.999 (0.756)
CC*	1.000 (0.928)
I/σI	22.09 (1.94)
Wilson B-factor	61.89
Completeness (%)	97.80 (95.73)
Redundancy	26.9 (23.0)
	
*Refinement*	
Resolution (Å)	2.50
No. of reflections (work/test)	107,541/1,512
*R*_work_/*R*_free_	22.07/23.9
No. of atoms	
Macromolecule	20,083
Ligand/ion	484
Water	59
B-factors	
Macromolecule	70.40
Ligand/ion	98.20
Water	61.10
r.m.s.d.	
bond lengths	0.003
bond angles	0.812

r.m.s., root mean square; r.m.s.d., root mean square deviation.
