# Supplementary material for: Structural basis of omalizumab therapy and omalizumab-mediated IgE exchange
Source: Nat Commun. 2016 May 19;7:11610. doi: 10.1038/ncomms11610 (PMC4873975; doi:10.1038/ncomms11610)
Supplement: Supplementary Information — Supplementary Figures 1-11 [file ncomms11610-s1.pdf]

**a**

| Omalizumab binding | $k_a$ ($M^{-1}s^{-1}$) | $k_d$ ($s^{-1}$) | $K_D$ (M) |
|---|---|---|---|
| IgE-$Fc_{3\text{-}4}$ (mammalian) | 1.55 *$10^{7}$ | 6.29*$10^{-4}$ | 4.05*$10^{-11}$ |
| IgE-R419N-$Fc_{3\text{-}4}$ (mammalian) | NB | NB | NB |
| IgE-$Fc_{3\text{-}4}$ (insect) | 3.21*$10^{7}$ | 1.52*$10^{-3}$ | 4.72*$10^{-11}$ |
| IgE-G335C-$Fc_{3\text{-}4}$ (insect) | 2.73*$10^{7}$ | 1.06*$10^{-3}$ | 3.89*$10^{-11}$ |
| IgE (Sus11) | 9.84*$10^{5}$ | 1.39*$10^{-4}$ | 1.42*$10^{-10}$ |

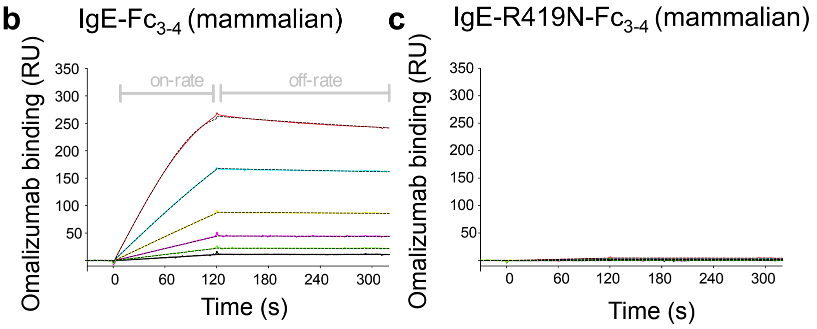


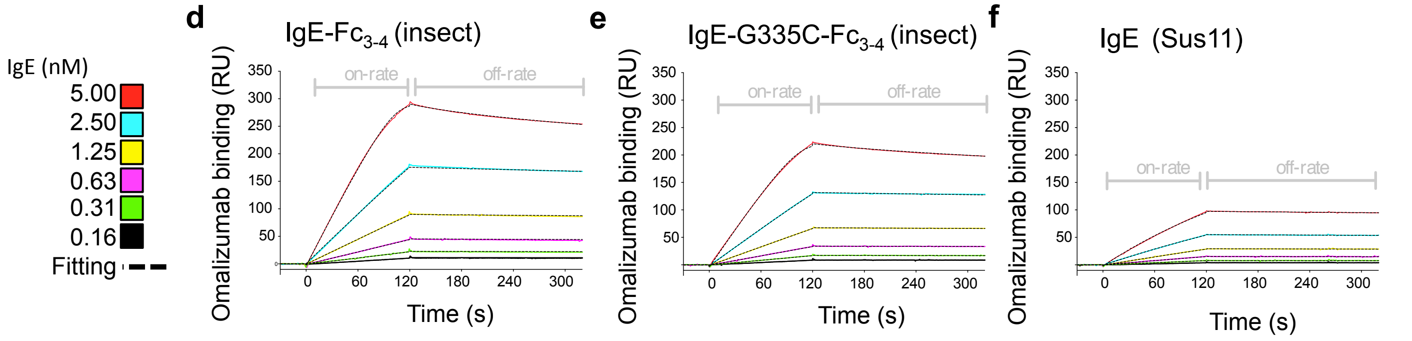


**Supplementary Figure 1: SPR sensorgrams and kinetic data for measured IgE:omalizumab interactions.** Omalizumab was immobilized on the sensor chip. The association and dissociation phases are labeled “on-rate,” and “off-rate,” respectively. **(a)** Summary kinetic data for all analytes. **(b)** Sensorgrams for mammalian derived IgE-$Fc_{3\text{-}4}$. **(c)** Sensorgrams for mammalian derived IgE-R419N-$Fc_{3\text{-}4}$. **(d)** Sensorgrams for insect derived IgE-$Fc_{3\text{-}4}$. **(e)** Sensorgrams for insect derived IgE-G335C-$Fc_{3\text{-}4}$. **(f)** Sensorgrams for hybridoma derived full-length IgE Sus11.

**a**

| FcεRIα binding | $k_a$ ($M^{-1}s^{-1}$) | $k_d$ ($s^{-1}$) | $K_D$ (M) |
|---|---|---|---|
| IgE-$Fc_{3\text{-}4}$ (mammalian) | $1.10*10^6$ | $1.02*10^{-3}$ | $9.33*10^{-10}$ |
| IgE-R419N-$Fc_{3\text{-}4}$ (mammalian) | $3.24*10^5$ | $7.732*10^{-4}$ | $2.26*10^{-9}$ |
| IgE-$Fc_{3\text{-}4}$ (insect) | $1.43*10^6$ | $9.46*10^{-4}$ | $6.60*10^{-10}$ |
| IgE-G335C-$Fc_{3\text{-}4}$ (insect) | NB | NB | NB |
| IgE (Sus11) | $3.35*10^6$ | $3.23*10^{-4}$ | $9.64*10^{-10}$ |

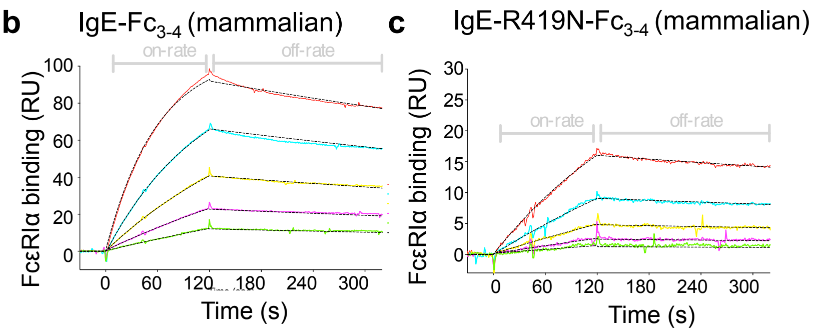


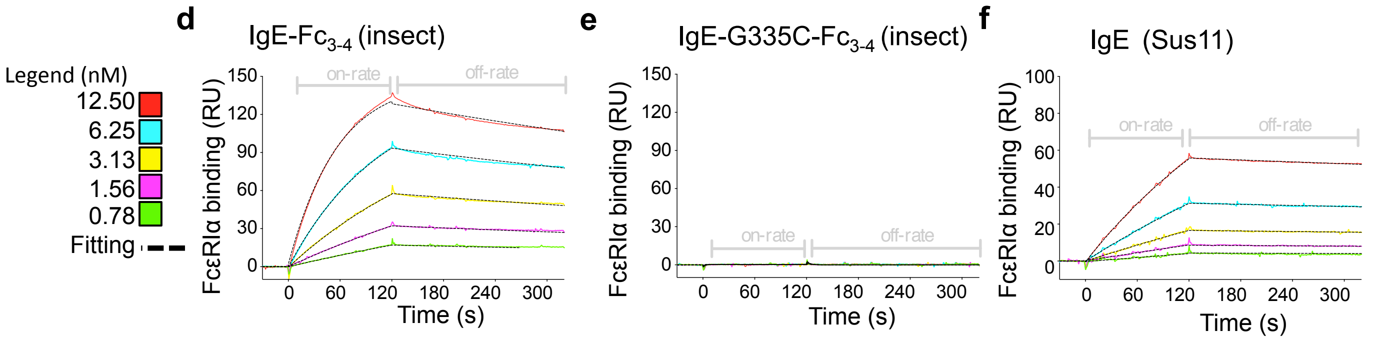


**Supplementary Figure 2: SPR sensorgrams and kinetic data for measured IgE:FcεRIα interactions.** FcεRIα was immobilized on the sensor chip. The association and dissociation phases are labeled "on-rate," and "off-rate," respectively. **(a)** Summary kinetic data for all analytes. **(b)** Sensorgrams for mammalian derived IgE-$Fc_{3\text{-}4}$. **(c)** Sensorgrams for mammalian derived IgE-R419N-$Fc_{3\text{-}4}$. **(d)** Sensorgrams for insect derived IgE-$Fc_{3\text{-}4}$. **(e)** Sensorgrams for insect derived IgE-G335C-$Fc_{3\text{-}4}$. **(f)** Sensorgrams for hybridoma derived full-length IgE Sus11.

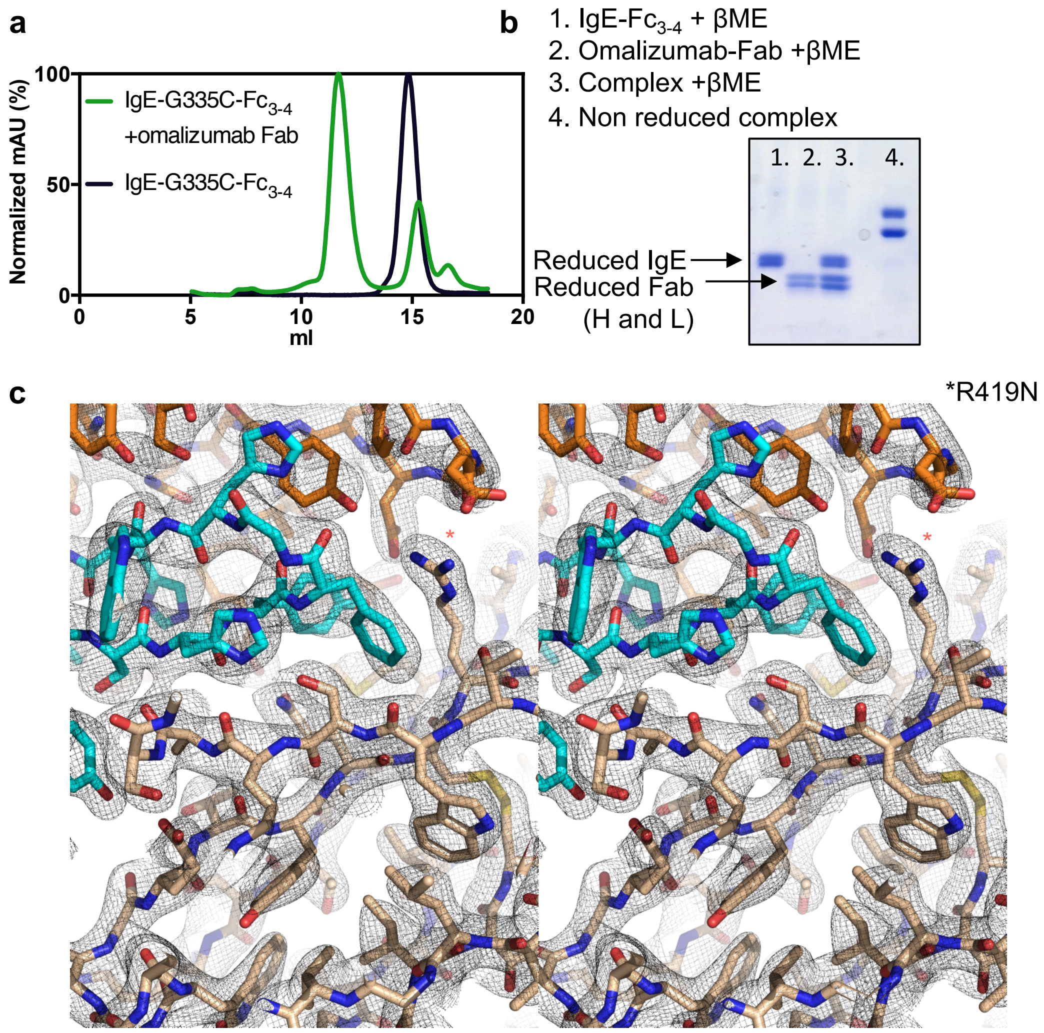


**Supplementary Figure 3: Preparation of IgE:omalizumab complexes and a representative electron density map of the IgE:omalizumab interface.** (**a**) Gel filtration of complex (*green*), with an excess of IgE, and IgE alone (*black)* (**b**) SDS-Page gel of purified and reduced IgE-G335C-$Fc_{3\text{-}4}$, omalizumab-Fab, and complex *(lanes 1-3)* with the non-reduced complex *(lane 4).* **(c)** Stereo image of the electron density from a SA composite omit 2mFo-DFc map contoured at 1σ near the IgE:omalizumab interface. The representation is colored by element, with IgE carbon atoms colored in tan, omalizumab heavy chain carbon atoms colored in cyan, and omalizumab light chain atoms colored in orange. The IgE residue R419N is labeled with a red asterisk.

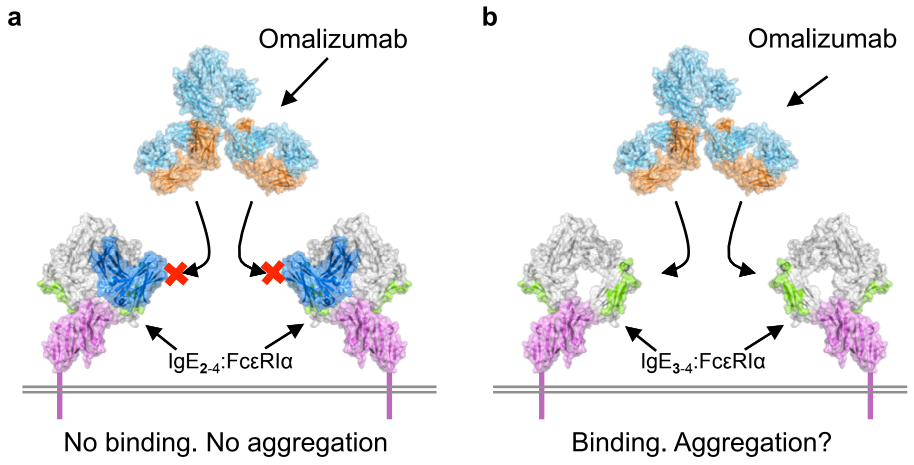


**Supplementary Figure 4: Cε2 obscures a omalizumab binding site.** (**a**) In the IgE-Fc$_{2-4}$:FcεRIα complex (2Y7Q) the IgE domain Cε2 (blue) and receptor FcεRIα (purple) obscure both omalizumab-binding sites (green). A schematic of two adjacent IgE-Fc$_{2-4}$:FcεRIα complexes on the cell surface reveals that cell bound IgE-Fc$_{2-4}$ could not be cross-linked by omalizumab. (**b**) A schematic of the truncated IgE-Fc$_{3-4}$:FcεRIα complex reveals that an omalizumab epitope in the IgE:FcεRIα complex is exposed, allowing binding to preformed complex and potentially omalizumab mediated cross linking of adjacent IgE:FcεRIα complexes. Omalizumab is depicted with the structure of a full-length mouse IgG1 antibody (1IG7) for schematic purposes only, with the heavy chain in blue and light chain in orange.

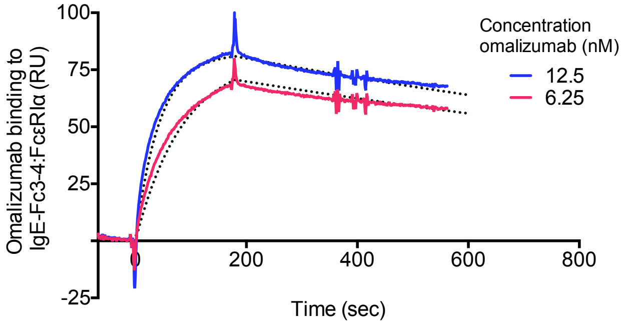


| Omalizumab binding | $k_a$ ($M^{-1}s^{-1}$) | $k_d$ ($s^{-1}$) | $K_D$ (M) |
|---|---|---|---|
| IgE-$Fc_{3-4}$:FcεRIα complex | 1.79 *$10^{6}$ | 5.60*$10^{-4}$ | 3.11*$10^{-10}$ |

**Supplementary Figure 5: Kinetic analysis of omalizumab binding to the IgE-$Fc_{3-4}$:FcεRIα complex.** Previously reported binding data (Ref #22 Eggel *et al.* Jaci 2014), was subject to kinetic analysis. FcεRIα was immobilized on the sensor chip, and loaded to a baseline RU with insect derived IgE-$Fc_{3-4}$ to generate the IgE-$Fc_{3-4}$:FcεRIα complex. The complex baseline runs from -67 to 0 seconds in the sensorgram. Omalizumab association is measured from 0-200 seconds, and dissociation is measured from 200-600 seconds. The data was fit with a 1:1 binding model, and the summary data is presented in the table.

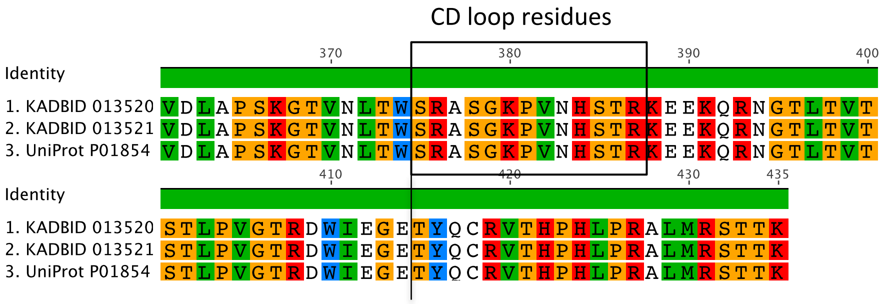


CD loop mutants from omalizumab binding studies: S407, R408, **S411**, **K415**, **N418**
Proposed correction: S407, R408, S410, K412, N415
Correction with IgE-G335C-$Fc_{3\text{-}4}$ numbering scheme: S375, R376, S378, K380, N383

**Supplementary Figure 6: Comparison of sequence variation across human IgE heavy chain sequence sources.** During validation of the omalizumab binding site, IgE heavy chain sequence variants and numbering schemes were corrected to allow for comparison across sources. Original studies with omalizumab employed sequences from 5$^{th}$ edition of the Sequences of Proteins of Immunological Interest. Two variants of the IgE heavy chain constant region (ID# 013520 and 013521) were aligned to the Uniprot P01854 sequence. This alignment revealed several minor regions of variation, yet alignment of regions including and surrounding the omalizumab epitope, revealed 100% homology across sources, and is displayed above. Despite correcting for minor sequence differences, and adjusting numbering schemes, 3 reported mutations from Presta *et al.* 2002 could not be reconciled with the source sequences. All of these mutations were reported to reside in the CD loop of IgE-Fc, and correspond perfectly with crystal data if the numbering for these residues is corrected as proposed.

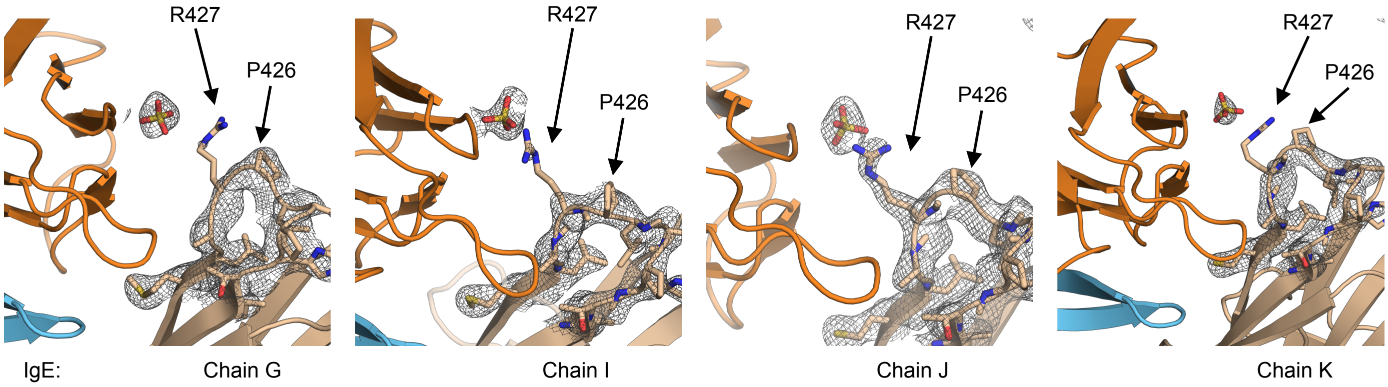


**Supplementary Figure 7: Electron density at IgE-R427 and IgE-P426.** Comparison of electron density in the region surrounding R427 and P426 in 2Fmo-DFc maps contoured to 1σ. Only the R427 residue within chain J has density accounting for the R427 side chain, preventing the placement of the R427 side chain in other NCS related IgE chains.

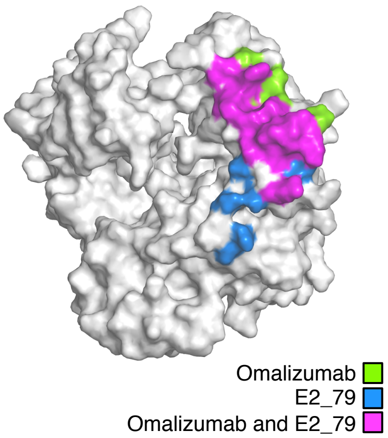


**Supplementary Figure 8: Overlap in binding footprint of E2_79 and omalizumab.** Comparison of the footprint of omalizumab and E2_79, as define by the residues with atomic contacts <4Å, reveals extensive overlap.

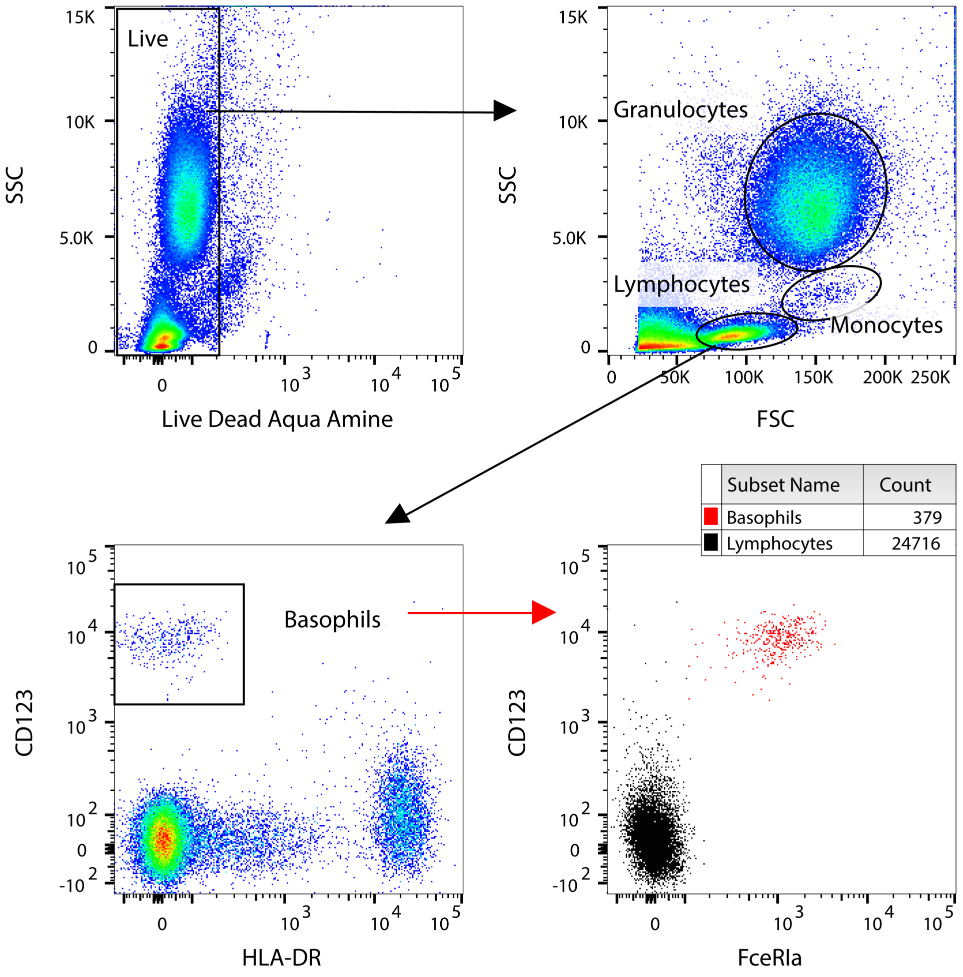


**Supplementary Figure 9: Gating scheme for basophils from peripheral blood.** Lymphocytes from the live cell population were selected by their SSC and FSC attributes, and subsequently CD123+ HLA-DR- cells were identified as human basophils. The basophils (red) as compared to the remainder of the lymphocyte population (black) also show strong staining for FcεRIα.

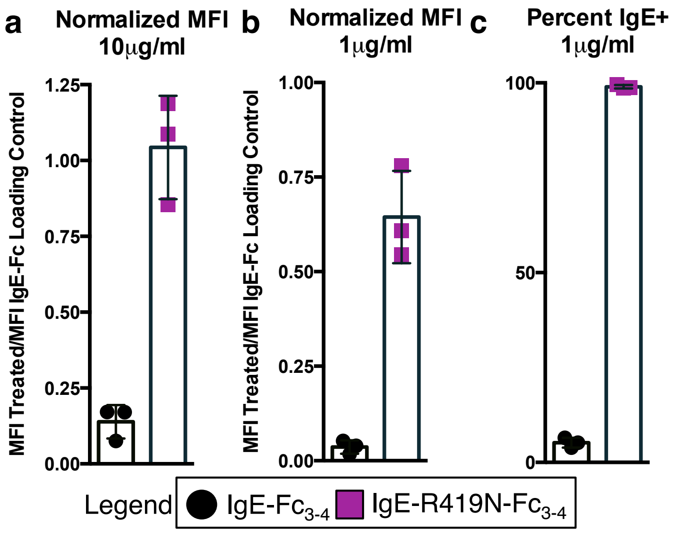


**Supplementary Figure 10: Summary data for IgE exchange.** (**a**) Overnight treatment of cells with omalizumab (25μM) and IgE-$Fc_{3\text{-}4}$ or IgE-R419N-$Fc_{3\text{-}4}$ (10 μg/ml or ~180nM) results in depletion of JW8-IgE, minimal exchange of IgE-$Fc_{3\text{-}4}$, and significant exchange for IgE-R419N-$Fc_{3\text{-}4}$ as assessed by the ratio of median fluorescent intensity of treated cells to IgE-$Fc_{3\text{-}4}$ loading controls within the same subject. (P= 0.0114 paired two-tailed T-test). (**b**) An identical analysis expressing the ratio of MFIs in cells treated with omalizumab (25μM) and IgE-$Fc_{3\text{-}4}$ or IgE-R419N-$Fc_{3\text{-}4}$ (1 μg/ml or ~18nM) reveals that virtually no repertoire exchange occurred in IgE-$Fc_{3\text{-}4}$ treated samples, while significant repertoire exchanged occurred in samples treated with IgE-R419N-$Fc_{3\text{-}4}$ (P=0.0119 paired two tailed T-test). This effect is more pronounced in the low dose samples when comparing populations of IgE positive cells, gating biotin-IgE-Fc untreated cells as the IgE negative population (**c**).

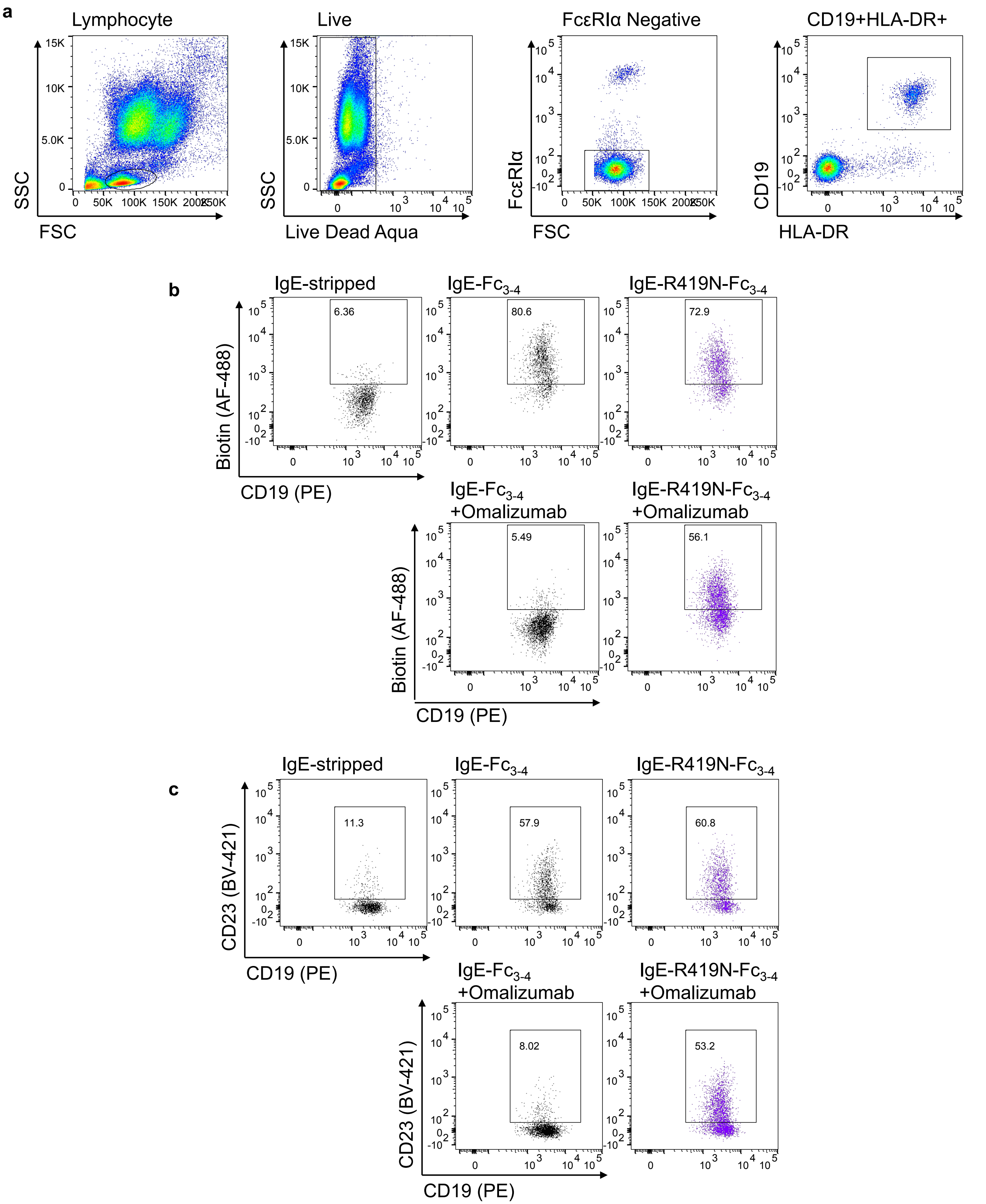
a
Lymphocyte
Live
FcεRIα Negative
CD19+HLA-DR+
SSC
FSC
Live Dead Aqua
FcεRIα
CD19
HLA-DR
b
IgE-stripped
IgE-Fc3-4
IgE-R419N-Fc3-4
6.36
80.6
72.9
Biotin (AF-488)
CD19 (PE)
IgE-Fc3-4 +Omalizumab
IgE-R419N-Fc3-4 +Omalizumab
5.49
56.1
c
IgE-stripped
IgE-Fc3-4
IgE-R419N-Fc3-4
11.3
57.9
60.8
CD23 (BV-421)
CD19 (PE)
IgE-Fc3-4 +Omalizumab
IgE-R419N-Fc3-4 +Omalizumab
8.02
53.2

**Supplementary Figure 11: IgE exchange on CD19+ HLA-DR+ B lymphocytes**. Representative plots of samples stained with B-cell markers CD19 and CD23 (N=2). (**a**) Representative gating of FcεRIα-, C19+HLA-DR+ B-lymphocytes (**b**) IgE-$Fc_{3-4}$ or IgE-R419N-$Fc_{3-4}$ staining of B-lymphocytes after IgE stripping, with IgE-$Fc_{3-4}$ or IgE-R419N-$Fc_{3-4}$ reloading, or after combination omalizumab and IgE-$Fc_{3-4}$ or IgE-R419N-$Fc_{3-4}$ treatment. (**c**) CD23 staining of B-lymphocytes after IgE stripping, with IgE-$Fc_{3-4}$ or IgE-R419N-$Fc_{3-4}$ reloading, or after combination omalizumab and IgE-$Fc_{3-4}$ or IgE-R419N-$Fc_{3-4}$ treatment. Note the apparent stabilization of surface CD23 in samples with high IgE-fc binding.
